# Long-term effects of flooding on mortality in England and Wales, 1994-2005: controlled interrupted time-series analysis

**DOI:** 10.1186/1476-069X-10-11

**Published:** 2011-02-02

**Authors:** Ai Milojevic, Ben Armstrong, Sari Kovats, Bridget Butler, Emma Hayes, Giovanni Leonardi, Virginia Murray, Paul Wilkinson

**Affiliations:** 1 Department of Social and Environmental Health Research, London School of Hygiene and Tropical Medicine, 15-17 Tavistock Place, London WC1H 9SH, UK; 2 Environment Agency, Bowbridge Close, Bradmarsh Business Park, Templeborough, Rotherham, South Yorkshire, S60 1BY, UK; 3 Centre for Radiation, Chemicals and Environmental Hazards, Health Protection Agency, 7th Floor, 330 High Holborn, London WC1V 7PP, UK

## Abstract

**Background:**

Limited evidence suggests that being flooded may increase mortality and morbidity among affected householders not just at the time of the flood but for months afterwards. The objective of this study is to explore the methods for quantifying such long-term health effects of flooding by analysis of routine mortality registrations in England and Wales.

**Methods:**

Mortality data, geo-referenced by postcode of residence, were linked to a national database of flood events for 1994 to 2005. The ratio of mortality in the post-flood year to that in the pre-flood year within flooded postcodes was compared with that in non-flooded boundary areas (within 5 km of a flood). Further analyses compared the observed number of flood-area deaths in the year after flooding with the number expected from analysis of mortality trends stratified by region, age-group, sex, deprivation group and urban-rural status.

**Results:**

Among the 319 recorded floods, there were 771 deaths in the year before flooding and 693 deaths in the year after (post-/pre-flood ratio of 0.90, 95% CI 0.82, 1.00). This ratio did not vary substantially by age, sex, population density or deprivation. A similar post-flood 'deficit' of deaths was suggested by the analyses based on observed/expected deaths.

**Conclusions:**

The observed post-flood 'deficit' of deaths is counter-intuitive and difficult to interpret because of the possible influence of population displacement caused by flooding. The bias that might arise from such displacement remains unquantified but has important implications for future studies that use place of residence as a marker of exposure.

## Background

The adverse health effects of floods are commonly assessed solely in terms of the deaths and injuries from drowning, electrocution or trauma and cases of infection that occur during, or very shortly after, the flood. In high income countries such as the UK, there are usually few immediate deaths except during the most exceptional flood events [1-4]. Economic and social costs can be very large, however, as floods such as those in the UK in the summer of 2006/2007 have shown [5].

Although these immediate impacts are usually limited, for many, being flooded is a traumatic experience, with potential to affect mental and perhaps physical well-being over the longer-term [6,7]. This may be especially so for groups such as the elderly, and those on low income or without adequate insurance cover.

One of the few studies that have examined the longer-term consequences of flooding was a controlled study of the Bristol floods of 1968 [8]. Its results suggested a 50% increase in all cause mortality among the flooded population in the 12 months following the flood (mortality fell in other areas), a similar rise in general practice attendance, and a doubling of hospital referrals and admissions. Such observation, together with consideration of the personal distress that flooding may cause to vulnerable individuals, raises the concern that the true health effects of flooding may be rather larger and long-lasting than statistics of immediate deaths and injuries suggest. To date, however, evidence about such effects remains very limited, despite their importance for public health policy in the context of climate change and pressures on land for residential development.

In this paper, we report our methodological insights from a large systematic analysis of mortality patterns in relation to all recorded floods in England and Wales from 1994 to 2005. We focused on mortality as death is very reliably recorded, and in the UK, routine mortality data include the postcode of residence which provides a high resolution marker of location for classifying whether an individual was flooded or lived close to a flood area. We interpret the available evidence as suggesting at least the possibility that mortality may be increased in the medium term by flooding.

## Methods

### Data

**Figure 1:**
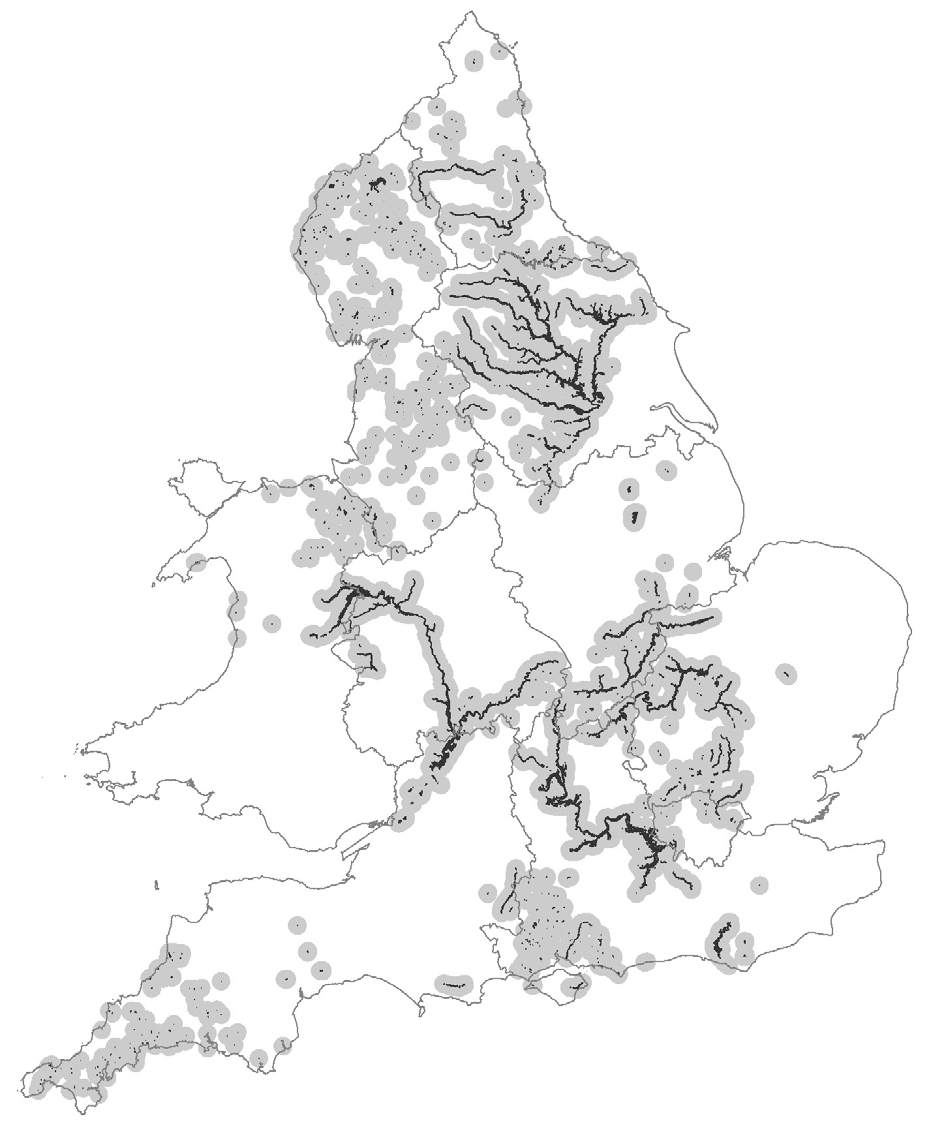
**Major recorded flood events, 1994-2005 (black filled) and 5 km boundary areas (gray filled)**.

The study was based on an analysis of mortality registrations, 1993-2006, linked by postcode of residence to the following data sets:

(i) The UK Environment Agency (EA)'s National Flood and Coastal Defence Database (NFCDD), Historic Flood Event Outlines, a Geographical Information System (GIS) data base of UK floods for 1994 to 2005, which contains data on the timing (date of onset) and geographical distribution of floods, as well as selected flood characteristics over this period (Figure 1), though there was no reliable marker of flood severity. (Depth of flood and even duration were too incomplete or unreliable to use.) These data were compiled from a combination of aerial photography, local survey, local authority records and other sources (see Additional File 1: Appendix Table 1 for recorded flood events and boundary sources).

(ii) Resident population and land area data for all 175,000 Output Areas (OAs) of England and Wales (data supplied by the Office for National Statistics (ONS)), together with an ONS marker of urban-rural classification [9]. An Output Area is the smallest geographical unit for which small-area census data are available. On average they contain 125 households (minimum of 40 resident households and 100 persons), and they are nested within Standard Table (ST) wards, which are those geographical areas (wards) for which the 2001 Census STs are available. Population density (persons per km^2^) was calculated by ST ward, and smoothed so that the smoothed population density was the average of the ward itself and of all OAs whose centroids lay within 5 km of the centroid of the ST ward.

(iii) The Index of Multiple Deprivation (IMD), 2004, for Lower Level Super Output Areas (LLSOAs). There are approximately 35,000 LLSOAs in England and Wales, each having an average of 1500 residents (97% have populations between 1000 and 2000). The IMD is a composite index of deprivation derived from data relating to the following domains: employment, health and disability, education skills and training, barriers to housing services, living environment and crime [10].

These data sets were linked to mortality registrations by the seven-digit (unit) postcodes of residence. The unit postcode on average relates to 14 households or around 40 individuals, and the coordinates of the postcode centroid are available to an accuracy of around 10 metres (to one metre in many urban areas), thus providing fairly precise localization of place of residence. Using data from the EA floods database on the extent of flood areas, postcodes were then classified with regard to flooding status as follows:

'Flooded (known date)' - postcodes flooded at any time for which a date of start of the flood (at least month) was recorded. For floods with date recorded to month only, we assigned the date of start of flooding to the first day of the month.

'Flooded (unknown date)' - postcodes flooded at any time for which either the year or month of start was not recorded.

'Adjacent' - each postcode within 5 km (10 km for selected analyses) of a flood boundary, with the date(s) of the start of flood(s) recorded where available.

Postcodes classified as 'Flooded (unknown date)', were excluded from analysis. Postcodes classified as 'Flooded (known date)' were categorized by application of three rules:

**Rule 1**. For the first recorded flood, deaths on each day in the year before the date of start of the first flood were considered pre-flood; deaths on each day in the year after the date of start of the first flood were considered post-flood.

**Rule 2**. For each subsequent flood, if the postcode was flooded (again) but more than two years after the date of start of the first (earlier) flood, **rule 1 **was repeated; if the postcode was flooded again, or was adjacent to, a flood starting less than two years after the date of start of the first (earlier) flood, deaths relating to the second period of flooding were not included in analysis, as the pre-flood period for the second flood might be affected by the post-flood changes of the first flood. However, we retained the analysis of the first period of flooding, as a second flood would be likely to accentuate the effect of change in deaths in the year after the first flood.

**Rule 3**. Deaths at postcodes occurring within 5 km of a flooded area were classified as 'adjacent' pre-flood or 'adjacent' post flood using the same dates as those used to classify postcodes in the corresponding flooded area into pre-flood and post-flood year.

### Analysis

Two analytical approaches were used.

#### (1) Controlled comparison of before-after change in mortality

The first and simplest approach compared the ratio of pre-flood year/post-flood year deaths in the flooded areas to the corresponding ratio for control (adjacent) areas within 5 km of the flood areas:

$$\begin{array}{l} Relative\;change\;in\;mortality\;(M)\\ =\frac{M{\left(t_{0},t_{0+1}\right)}_{flooded}/M{\left(t_{0-1},t_{0}\right)}_{flooded}}{M{\left(t_{0},t_{0+1}\right)}_{adjacent}/M{\left(t_{0-1},t_{0}\right)}_{adjacent}}, \end{array}$$

where *t_0 _*= date of onset of the flood, *t_0-1 _*= the date one year before *t_0_*, and *t_0 + 1 _*= the date one year after the onset of the flood.

**Figure 2:**
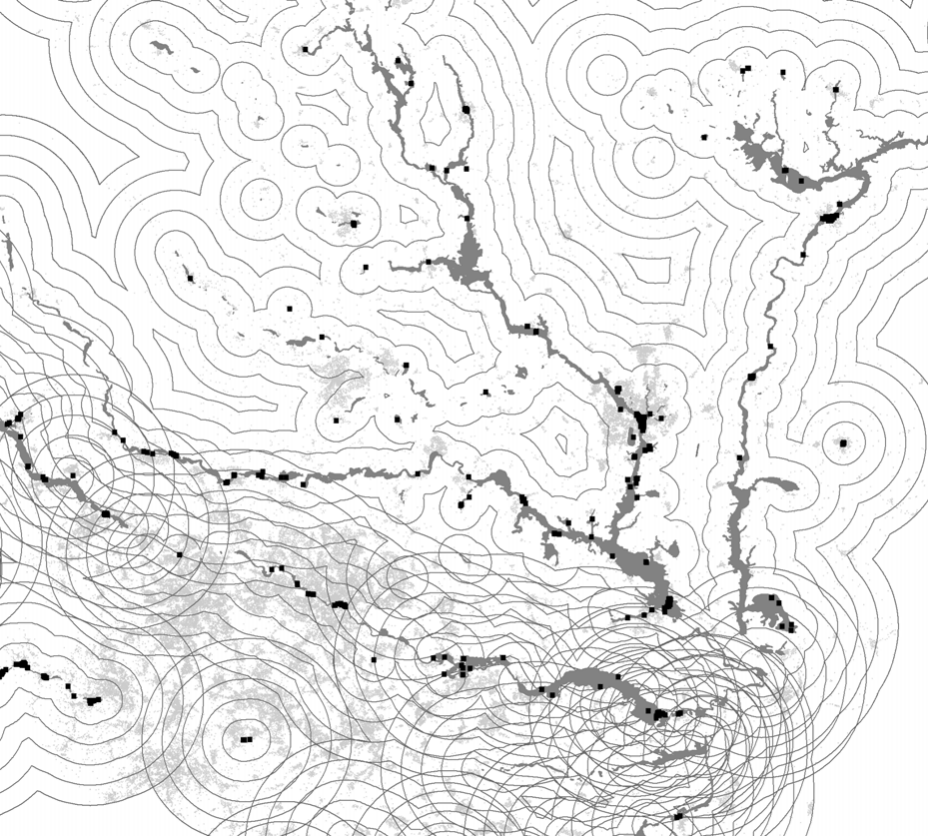
**GIS generated 'map' of a local area illustrating the definition of boundaries of 0-, 2-, 4-, 6-, and 8-10 km (gray lines) around flood areas (dark gray filled boundaries)**. Postcodes in flood areas are shown as black squares, those in buffering-boundary areas as light gray small dots.

Further tabulations were then done by five distance bands around the flooded area defined by the following distance boundaries from the perimeter of the flood area: 0-, 2-, 4-, 6-, 8-10 km (Figure 2).

Results were stratified by age-group (0-64, 65-74, 75-84, 85+ years), sex, International Classification of Diseases, 9^th ^and 10^th ^revisions (ICD-9, ICD-10) cause of death, urban rural status, quintile of the Index of Multiple Deprivation score for the LLSOA of residence, and place of death as recorded on the death certificate. The cause of death groups analysed were: Infectious disease (ICD-9 001-139.8, ICD-10 A00-B99); cardiovascular disease (ICD-9 390-459, ICD-10 I00-I99); respiratory disease (ICD-9 460-519, ICD-10 J00-J99); mental illness (ICD-9 290-319, ICD-10 F00-F99); external causes (ICD-9 800-999, ICD-10 V01-Y99); and all others.

#### (2) Analysis of observed/expected deaths in flooded areas

**Figure 3:**
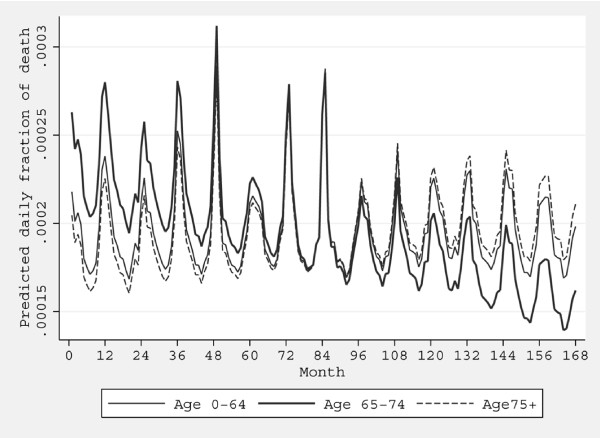
**Predicted daily fractions of death over time in study period by age-group**. Results are shown for men, Northern East, the lowest deprivation quintile, highest quintile of population density. Month 0 = January 1993, month 168 = December 2006.

The second approach entailed calculation of the expected number of deaths in flooded areas based on analysis of trends over time in population counts of deaths. Analyses of trends in deaths (rather than rates) was used because accurate year by year population denominators at the very small area level needed to define flooded populations are not available in the UK, For each postcode classified as "flooded", the expected pattern of deaths was determined for the study period, 1993 to 2006, by analysis of the national pattern of death counts in never-flooded areas stratified by date, age-group (0-64, 65-74, 75+ years), sex, region, quintile of the 2004 IMD score, and population density (quintile 1 (most rural) *vs *quintiles 2-5). The pattern of deaths over time was fitted using conditional Poisson regression models that included interactions of elapsed month with region, age-group, sex, deprivation group and population density (see Additional File 1: Appendix Table 4 for modification of covariates over time in study period). The fitted model parameters were then used to compute for each "flooded" postcode an expected death count for each day in the interval t_0-1 year _to t_0+1 year_, constructed so that the total expected deaths equal the total observed deaths. An example of the pattern of expected deaths by date for one region and socio-demographic group is shown in Figure 3.

It should be noted that both methods of analysis control for the effect of year to year variations in winter or influenza mortality (which could theoretically deplete the pool of susceptible people), as both methods entail comparisons of the pre-post change in morality in flooded areas with that in non-flooded control populations over the same time periods.

Ethics approval for this work was granted by Ethics Committee of the London School of Hygiene and Tropical Medicine.

## Results

For the study period 1994 to 2005, the EA floods data base recorded 319 flood events (8,886 separate flood boundaries), covering a total flooded land area of 1,092 km^2 ^(1,679 km^2 ^ignoring overlaps of separate flood areas). The areas adjacent to (<5 km from the boundary of) flooded areas covered approximately 48,044 km^2^. Of the 1,824,113 postcodes for England and Wales in the November 2007 National Statistics Postcode Directory, the centroids of 4,083 (0.2%) fell within flood areas, 729,092 (40%) were within 5 km of a flood area, and 1,090,938 (59.8%) were ≥5 km of a flood area. Seventeen percent of the postcodes in flooded areas recorded only the month of onset of flooding, and for these the start date was assigned to the first day of the month. We estimated that flooded areas affected around 57,000 households or approximately 237,000 persons at some point over the study period. See Additional File 1: Appendix Table 2 for a comparison of the characteristics of the populations in flooded, adjacent and other areas.

### Before-after change in flooded and adjacent areas

Over the study period as a whole, a total of 771 deaths occurred in the year before a flood with known date of onset; there were 693 deaths at the same postcodes in the year after the onset of flooding, a relative reduction of 10% (Table 1). In adjacent areas within 5 km of the flooded areas, mortality was just one percent lower in the post-flood year compared with the pre-flood year. Thus, the ratio of the relative change in mortality in flooded areas to that in adjacent areas was 0.90 (0.82, 1.00), indicating a relative 'deficit' of deaths in flooded areas in the year after flooding compared with adjacent areas.

**Table 1:** Deaths in flooded and non-flooded areas in the year before and after the flood onset

	Flooded area			Non-flooded boundary area ≤5 km			Ratio (95%CI) of change in flooded/non-flooded areas [(B/A)/(D/C)]
	
	Year before flood (A)	Year after flood (B)	Ratio (B/A)	Year before flood (C)	Year after flood (D)	Ratio (D/C)	
All areas	771	693	0.90	286793	285289	0.99	0.90 (0.82 - 1.00)
Age (years)							
0-64	136	127	0.93	49437	48724	0.99	0.95 (0.74 - 1.21)
65-74	137	118	0.86	57451	55117	0.96	0.92 (0.72 - 1.18)
75-84	234	204	0.87	95045	94352	0.99	0.87 (0.72 - 1.05)
85+	264	244	0.92	84860	87096	1.03	0.88 (0.73 - 1.05)
Sex							
Men	369	332	0.90	136511	135225	0.99	0.90 (0.78 - 1.05)
Women	402	361	0.90	150282	150064	1.00	0.89 (0.77 - 1.03)
Cause of death							
Infectious	2	7	3.60	1885	2056	1.09	3.10 (0.70 - 13.8)
Cardiovascular	311	289	0.89	113725	113138	0.99	0.92 (0.79 - 1.09)
Respiratory	116	104	0.90	46565	41569	0.89	0.97 (0.74 - 1.27)
Mental illness	23	20	0.87	5798	6648	1.15	0.74 (0.41 - 1.35)
External	20	28	1.40	8682	8768	1.01	1.41 (0.79 - 2.52)
All others	299	245	0.82	110139	113110	1.03	1.03 (0.81 - 0.96)
Urban/rural							
Urban	470	428	0.91	233651	232068	0.99	0.91 (0.80 - 1.04)
Rural	301	265	0.88	53142	53221	1.00	0.88 (0.74 - 1.04)
Deprivation quintile							
Q1 (least deprived)	205	179	0.87	57222	57161	1.00	0.87 (0.71 - 1.07)
Q2	176	166	0.94	57306	57165	1.00	0.90 (0.72 - 1.11)
Q3	193	183	0.95	57261	57003	1.00	0.97 (0.79 - 1.19)
Q4	133	120	0.90	57217	57171	1.00	0.89 (0.70 - 1.14)
Q5 (most deprived)	64	45	0.70	57787	56789	0.98	0.74 (0.51 - 1.09)
Place of death							
Home	159	132	0.83	56215	54866	0.98	0.84 (0.67 - 1.06)
Hospital	395	353	0.89	161496	161064	1.00	0.88 (0.77 - 1.02)
Hospice	43	30	0.70	13024	13054	1.00	0.71 (0.45 - 1.15)
Nursing home	84	83	0.99	27387	27975	1.02	0.95 (0.70 - 1.30)
Residential home	71	65	0.92	21067	20597	0.98	0.90 (0.64 - 1.26)
Other	19	30	1.58	7604	7733	1.02	1.53 (0.85 - 2.76)

There was little evidence that this relative deficit varied with age-group or sex, or with urban-rural status, or with quintile of socio-economic deprivation (Table 1). Although point estimates suggested a relative increase in post-flood deaths in flooded areas from infectious and external causes, the confidence intervals were compatible with a deficit for all the ICD sub-groups examined. Nor was there indication of variation by place of death, all categories of which yielded point estimates of deficit except for the comparatively small number of deaths occurring at places other than at home, hospital, hospice, nursing home or residential home.

The pattern of relative deficit of deaths in the year after flooding was also seen when analysis was confined to floods occurring in the single year 2000 (a year of many floods across the UK) - Table 2. For that year, each of the five defined distance bands around the flood area (0-, 2-, 4-, 6-, 8-10 km) showed small decreases in deaths in the post-flood year, while the flooded area itself showed a deficit of 19% compared with the pre-flood year: this yielded a ratio of pre-to-post-flood change in deaths for flood areas of 0.87 (95% CI 0.74, 1.04) by comparison with the outermost distance band. Results for other analyses by distance band are presented in the Additional File 1: Appendix Table 3 for before-after change ratio relative to flooded areas by distance band by sub-groups).

**Table 2:** Deaths in the year before and after flood onset by distance from the flood area: floods occurring in 2000 only

Area	Before (A)	After (B)	Ratio (B/A)	Before-after change ratio (95%CI) relative to 8-10 km	Before-after change ratio (95%CI) relative to flooded areas
Flooded areas	332	269	0.81	0.87 (0.74 - 1.04)	1.00
Non-flooded boundary areas					
0 - 2 km	64358	61962	0.96	1.01 (0.99 - 1.03)	1.16 (0.99 - 1.37)
2 - 4 km	50601	49278	0.97	1.01 (0.99 - 1.03)	1.22 (1.03 - 1.43)
4 - 6 km	45052	43721	0.97	1.00 (0.98 - 1.03)	1.19 (1.01 - 1.40)
6 - 8 km	37525	36624	0.98	1.01 (0.98 - 1.03)	1.21 (1.02 - 1.43)
8 - 10 km	33547	32334	0.96	1.00	1.14 (0.96 - 1.36)

### O/E deaths in flooded areas

**Figure 4:**
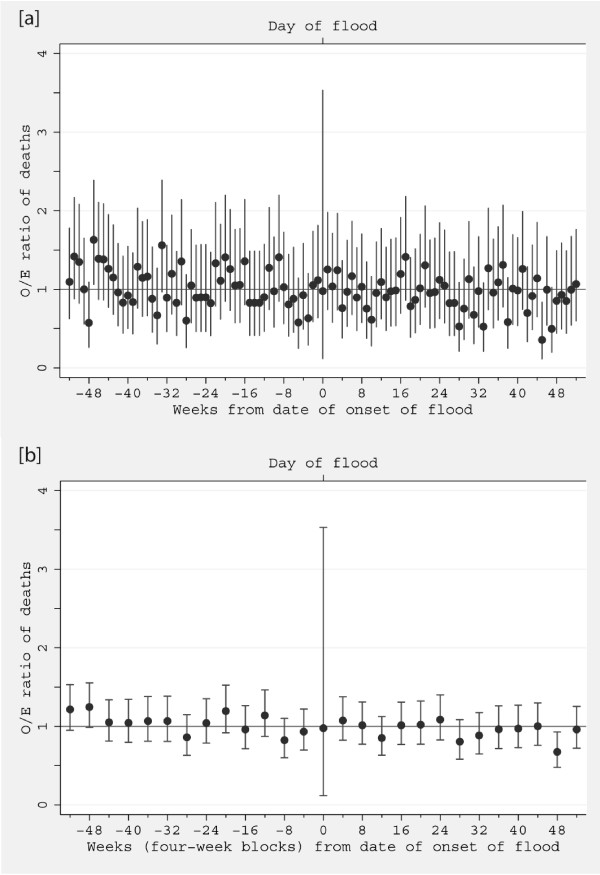
**[a] Ratio of observed to expected deaths by week from date of onset of flooding; [b] same data shown by four-week blocks**. Note the suggestion of a step change after 24 weeks post-flood.

Analyses of the ratio of observed (O) to expected (E) deaths in flooded areas also support the finding of a deficit of deaths in the year after flooding compared with the year before flooding (Table 3). Across all year combined, the O/E ratio was 1.05 for flooded areas in the pre-flood year, and 0.95 for the post-flood year, an overall relative deficit of 0.90 (Table 3). Although there was some variation by month in the O/E ratio, there was not a convincing pattern of an initial excess of deaths in the first few months after the flood: although five of the first six four-weekly O/E ratios were above 1.0 in the post flood period, all six of the corresponding ratios for the year before the flood were also above 1.0. The post-flood deficit, however, was largely confined to the second half of the post-flood year. Neither did examination of the flood area O/E ratios by individual week (Figure 4) reveal any clear pattern of variation by time from the flood event.

**Table 3:** Observed and expected number of deaths in flooded areas by four week periods from the date of onset of flooding

Weeks from date of onset of flood	Observed deaths (O)	Expected deaths (E)	O/E ratio (95%CI)	**Ratio of deaths **^**a**^
Year before flood				
-52 to -49	72	59.25	1.22 (0.95 - 1.53)	-
-48 to -45	79	63.40	1.25 (0.99 - 1.55)	-
-44 to -41	66	62.76	1.05 (0.81 - 1.34)	-
-40 to -37	60	57.48	1.04 (0.80 - 1.34)	-
-36 to -33	58	54.33	1.07 (0.81 - 1.38)	-
-32 to -29	57	53.38	1.07 (0.81 - 1.38)	-
-28 to -25	46	53.42	0.86 (0.63 - 1.15)	-
-24 to -21	56	53.80	1.04 (0.79 - 1.35)	-
-20 to -17	64	53.61	1.19 (0.92 - 1.52)	-
-16 to -13	51	53.06	0.96 (0.72 - 1.26)	-
-12 to -9	61	53.53	1.14 (0.87 - 1.46)	-
-8 to -5	45	54.69	0.82 (0.60 - 1.10)	-
-4 to -1^b^	53	56.84	0.93 (0.70 - 1.22)	-
Pre-flood total (weeks -52 to -1) ^c^	768	729.55	1.05 (0.98 - 1.13)	1.00

Year after flood				
Day of flood	2	2.05	0.98 (0.12 - 3.53)	0.93
1 to 4 ^c^	62	57.73	1.07 (0.82 - 1.38)	1.02
5 to 8	59	58.11	1.02 (0.77 - 1.31)	0.96
9 to 12	50	58.60	0.85 (0.63 - 1.12)	0.81
13 to 16	58	57.28	1.01 (0.77 - 1.31)	0.96
17 to 20	57	55.87	1.02 (0.77 - 1.32)	0.97
21 to 24	59	54.34	1.09 (0.83 - 1.40)	1.03
25 to 28	43	53.34	0.81 (0.58 - 1.09)	0.77
29 to 32	47	53.19	0.88 (0.65 - 1.18)	0.84
33 to 36	52	54.08	0.96 (0.72 - 1.26)	0.91
37 to 40	54	55.53	0.97 (0.73 - 1.27)	0.92
41 to 44	57	56.89	1.00 (0.76 - 1.30)	0.95
45 to 48	38	56.19	0.68 (0.48 - 0.93)	0.64
49 to 52	54	56.17	0.96 (0.72 - 1.25)	0.91
Post-flood total (weeks 0 to 52) ^c^	692	729.36	0.95 (0.88 - 1.02)	0.90

^a ^Ratio of deaths in post-flood periods compared with average for the preceding year.

^b ^Week 1 begins on the day after the flood, i.e. T_0+1 day_

^c ^The numbers of total observed deaths differ slightly from those in Table 1 because the four-week block include an additional day either side of the date of onset of the flood.

## Discussion

This is one of the largest, systematic studies of mortality patterns in relation to floods published to date. Its findings are counter-intuitive, however, and its interpretation unclear primarily because of the unexpected observation of an apparent 'reduction' in mortality in flood areas in the year after flooding. The principal question is whether this apparent reduction is a true effect or artefact.

The study capitalized on the particular advantage of the UK in having a high resolution geographical marker for place of residence for all mortality registrations, namely the home postcode, which is specific to an average of around 14 households, coupled with a national data base of flood events compiled over many years by the Environment Agency. With the use of GIS technology, we were able to 'overlay' postcode and flood area maps, and so classify deaths according to their geographical and temporal position with respect to the many floods that have occurred in England and Wales since 1994. Our focus on mortality was motivated firstly by the observation of the apparently large increases in mortality and other health outcomes in the year after the 1968 Bristol floods [8], and secondly by the reliability of mortality data which allowed testing of detailed methods for analysis of spatio-temporal patterns of change in relation to flood events. There were important limitations, however, including that we did not have reliable data on flood severity, only on the fact of flooding. This means that homes with the more severe forms of flooding (as characterized by depth, duration or suddenness of flooding, for example) were not separately identifiable for potentially important subgroup analyses. However, even short-lived low level flooding is still damaging and extremely disruptive. A further limitation is that the analyses were based on recorded postcode of residence, rather than on tracking of individuals who had been flooded, and this may have had important implications for the recording of subsequent mortality among the flooded population, as described below.

At face value, far from showing a flood-related increase in mortality, the results suggest that flooding is associated with around a 10% decline in mortality in the flooded population over the following year. The apparent 'deficit' of deaths has several possible explanations. Artefact of the analytical methods seems unlikely because of the similarity of results achieved through two different analytical approaches: both the before-after comparison of mortality in flooded against boundary areas and the calculation of the observed/expected ratio of deaths within flood areas produce similar findings. Note that, although we did not have population denominators, our analyses do not assume constant populations over time, only that there is no systematic difference in the change in populations in flooded and non-flooded areas over the many hundreds of flood locations and events analyses.

A second possibility is artefact arising from the data, specifically the ascertainment of mortality, which may be incomplete for flooded households if evacuation leads to some deaths being registered at different addresses from their usual place of residence. This bias would be particularly great if the frail (and thus most at risk of dying) were the ones most likely to be evacuated from flooded areas. In England and Wales, at death registration, it is up to the informant to decide what address to give as the usual place of residence. It is possible, therefore, that a proportion of deaths occurring among evacuated families could be registered to their temporary rather than their original address. If so, we should expect the deficit of deaths to be greatest in the immediate post-flood period, and gradually to return to normal. Data from the Department of Communities and Local Government [11] suggest that, nationally, around 13% of flooded households were still displaced from their own homes six months after the flood, often to more than a mile from the flooded area [12,13], but that only around 4% were still displaced by one year. If representative, this pattern and timing of displacement seems unlikely to be able to turn an appreciable mortality excess, such as that observed by Bennet, into our observed 10% deficit, suggesting that mortality impacts over the last decade or so have been much smaller than they were in Bristol in 1968, and perhaps insubstantial overall. It is possible that the response to flooding and the population's resilience to its adverse impacts may have changed appreciably over time.

A third possible explanation is one of true effect - namely that being flooded brings a level of attention from health and social services and from friends and relatives that has a positive effect on well-being and reduces mortality. This possibility is of course entirely speculative, and without any direct evidence, but it cannot be disregarded until there is clearer evidence to demonstrate that the post-flood deficit of deaths observed in our analyses is the result of an artefact of evacuation or other bias. Further work is now being undertaken to assess how such uncertainties might be addressed.

What we had expected to find in our study was either no significant change in mortality after flooding or a rise directly or indirectly attributable to the disruption of the flood and possible effects on mental well-being. Flood disasters, like other traumatic events, appear to be associated with increased rates of the common mental disorders (CMD), anxiety and depression [6], and indeed the psychological sequelae of floods may outweigh other forms of flood-related morbidity in settings such as the UK. Qualitative research indicates the psychological effects are exacerbated by the loss of valuable and cherished possessions, and the stress associated with evacuation, cleaning, dealing with insurance claims and organising repairs and refurbishment [14,15], A study of the 1974 floods in Brisbane found that incidence of CMD was directly related to dissatisfaction with help received [16]. Given the consistently high correlation between physical and psychiatric morbidity, it remains possible that increased rates of CMD may contribute to both higher rates of medical consultation following a flood and to increased mortality.

Studies of the effect of hurricane Katrina provide direct evidence that severe flooding may carry substantial health burdens, including effects on longer-term mortality [17-19]. This high profile event may be qualitatively different in character from the many more modest floods that were analysed in our England and Wales study. Nonetheless, they emphasize the potential for substantial adverse health impacts of flooding which is important to understand for developing effective public health responses and for informing cost-benefit aspects of decisions about flood protection measures, including the use of flood-prone land for building and the value of adaptations. Not since the major coastal floods of 31 January to 1 February 1953 have large numbers of people in the UK died directly as a result of flooding [20,21], but the question of unnoticed long-term impacts on health remains unresolved, and deserves further study.

## Conclusions

High-resolution geo-coded mortality and other routine health datasets in the UK have provided the possibility to examine long-term health effects of floods, but our first experience with analysis of mortality data has raised important questions about potential bias, mainly in relation to recording of place of death in flood-displaced populations, that limits interpretation. Our results do not provide evidence that floods in England and Wales have been associated with a rise in mortality in the year after flooding, but further enquiry is needed to address key uncertainties.

## Abbreviations

CMD: Common Mental Disorders; E: Expected; EA: Environment Agency; GIS: Geographical Information System; ICD: International Classification of Diseases; IMD: The index of Multiple Deprivation; LLSOAa: Lower Level Super Output Areas; O: Observed; OA: Output Areas; O/E ratios: Observed/Expected ratios; ONS: Office for National Statistics; ST wards: Standard Table wards; UK: United Kingdom

## Competing interests

The authors declare that they have no competing interests.

## Authors' contributions

AM carried out statistical analysis and drafted the manuscript. BA helped statistical analysis. SK, GL, VM, and PW conceived of the study and participated in its design and coordination. BB and EH made substantial contributions to acquisition of the NFCDD data. All authors read and approved the final manuscript.

## Supplementary Material

Additional File 1 **Appendix tables**. Appendix Table 1. National Flood and Coastal Defence Database (NFCDD), Historic Flood Ebent Outlines: (a) recorded flood events in England and Wales, 1994-2005, (b) boundary sources used in defining the flood event outlines. Appendix Table 2. Characteristics of flooded area and adjacent to flooded area. Appendix Table 3. Death in the year before and after flood onset by boundary distance band from the flooded area: floods occurring in 2000 only by age, sex, urban/rural, cause of death, population density, place of death, region, and deprivation quintile. Appendix Table 4. Modification of covariates over time (10 years) in study period. Click here for file
