# Supplementary material for: Long-term effects of flooding on mortality in England and Wales, 1994-2005: controlled interrupted time-series analysis
Source: Environ Health. 2011 Feb 2;10:11. doi: 10.1186/1476-069X-10-11 (PMC3042000; doi:10.1186/1476-069X-10-11)
Supplement: Additional File 1 — Appendix tables. Appendix Table 1. National Flood and Coastal Defence Database (NFCDD), Historic Flood Ebent Outlines: (a) recorded flood events in England and Wales, 1994-2005, (b) boundary sources used in defining the flood event outlines. Appendix Table 2. Characteristics of flooded area and adjacent to flooded area. Appendix Table 3. Death in the year before and after flood onset by boundary distance band from the flooded area: floods occurring in 2000 only by age, sex, urban/rural, cause of death, population density, place of death, region, and deprivation quintile. Appendix Table 4. Modification of covariates over time (10 years) in study period. [file 1476-069X-10-11-S1.PDF]

Appendix Tables

Appendix Table 1. National Flood and Coastal Defence Database (NFCDD), Historic Flood Event Outlines

(a) Recorded flood events in England and Wales, 1994-2005

Year	Events (N)	Boundary areas (N)	Total flooded area (km^2)	Most damaged regions[a] (km^2)
1994	8	13	2.36	SW(2)
1995	17	455	179.65	YH(152), NE(25), NW(2)
1996	8	11	0.69	
1997	13	25	5.50	NW(5)
1998	23	895	393.49	WM(128), EE(84), EM(79), SE(47), W(37), SW(18)
1999	19	86	40.41	YH(39)
2000	101	3263	712.92	YH(250), WM(153), SW(136), SE(92), W(44), EE(13), EM(12), NW(4), LON(4), NE(4)
2001	7	315	26.73	EE(26)
2002	26	2864	198.21	SE(136), W(34), WM(22), NW(3), LON(2)
2003	8	86	7.71	EE(5), SW(2)
2004	28	666	60.33	WM(37), W(21), SW(1)
2005	27	207	52.16	NW(21), NW(20), YH(11)
1994-2005	285	8886	1680.16	YH(452), WM(340), SE(277), SW(160), W(137), EE(129), EM(93), NE(49), NW(36), LON(6)

[a] NE: North East, NW: North West, YH: Yorkshire and the Humber, EM: East Midlands, WM: West Midlands, E: East, LON: London, SE: South East, SW: South West. Regions are shown only ≥ 1km^2.

(b) Boundary sources used in defining the flood event outlines, NFCDD (1994-2005).

Boundary Source	Outlines	(%)
Aerial photography	3,977	45%
Survey (Agency)	2,676	30%
Survey (consultants)	702	8%
Visual	239	3%
Local Authority	83	1%
Public	48	1%
Memory	6	0%
Other	643	7%
Unknown	512	6%
Total	8,886	100%

Appendix Table 2. Characteristics of flooded area and adjacent to flooded area.

Area	Postcodes[1]		Deprivation group (%)[2]					Urban area (%)[3]
	N	%	Q1	Q2	Q3	Q4	Q5	
Flooded area	4814	0.2%	22.5	23.5	20.2	22.3	11.5	62.0
Adjacent area	863,764	44.1%	21.6	19.0	18.2	19.9	21.3	78.6
Other area	1,090,335	55.7%	18.7	20.8	21.5	20.1	19.0	71.8
All	1,958,913	100.0%	20.0	20.0	20.0	20.0	20.0	74.8

[1] N of postcodes represents population size: 14 households per postcode on average

[2] Quintile of Index of Multiple Deprivation 2004 score: Q1 (least deprived) – Q5 (most deprived)

[3] Area >10k population (ONS urban/rural category)

Appendix Table 3. Deaths in the year before and after flood onset by boundary distance band from the flooded area: floods occurring in 2000 only by age, sex, urban/rural, cause of death, population density, place of death, region, and deprivation quintile.

	Boundary Area		Before (A)	After(B)	Ratio (B/A)	Before-after change ratio (95%CI) relative to 8-10 km		Before-after change ratio (95%CI) relative to flooded areas	
**Age**									
0-64	Flooded		65	49	0.75	0.75	(0.50-1.12)	1.00	
	Non-flooded	0-2km	10,885	10,372	0.95	0.95	(0.91-1.01)	1.24	(0.85, 1.82)
		2-4km	8,893	8,823	0.99	0.99	(0.94-1.04)	1.27	(0.87, 1.85)
		4-6km	7,686	7,493	0.97	0.98	(0.93-1.03)	1.31	(0.89, 1.92)
		6-8km	6,539	6,255	0.96	0.95	(0.91-1.01)	1.24	(0.84, 1.82)
		8-10km	5,899	5,891	1.00	1.00		1.33	(0.89, 1.98)
65-74	Flooded		62	41	0.66	0.75	(0.50-1.13)	1.00	
	Non-flooded	0-2km	12,533	11,659	0.93	1.01	(0.96-1.06)	1.33	(0.90, 1.99)
		2-4km	10,187	9,402	0.92	0.98	(0.94-1.03)	1.40	(0.94, 2.09)
		4-6km	8,895	8,329	0.94	0.99	(0.94-1.04)	1.38	(0.93, 2.06)
		6-8km	7,231	6,882	0.95	1.00	(0.95-1.05)	1.40	(0.93, 2.10)
		8-10km	6,398	6,099	0.95	1.00		1.33	(0.76, 1.36)
75-84	Flooded		99	79	0.80	0.90	(0.73-1.31)	1.00	
	Non-flooded	0-2km	21,383	20,605	0.96	1.04	(1.00-1.08)	1.17	(0.87, 1.58)
		2-4km	16,609	16,383	0.99	1.04	(1.00-1.08)	1.26	(0.93, 1.71)
		4-6km	14,666	14,350	0.98	1.04	(1.00-1.08)	1.25	(0.91, 1.70)
		6-8km	12,358	12,215	0.99	1.04	(1.00-1.08)	1.27	(0.93, 1.74)
		8-10km	10,943	10,295	0.94	1.00		1.12	(0.76, 1.36)
85+	Flooded		106	100	0.94	0.98	(0.73-1.31)	1.00	
	Non-flooded	0-2km	19,557	19,326	0.99	1.00	(0.96-1.04)	1.05	(0.90, 1.39)
		2-4km	14,912	14,670	0.98	1.02	(0.98-1.06)	1.04	(0.79, 1.38)
		4-6km	13,805	13,549	0.98	0.99	(0.96-1.03)	1.02	(0.77, 1.34)
		6-8km	11,397	11,272	0.99	1.01	(0.97-1.05)	1.10	(0.82, 1.46)
		8-10km	10,307	10,049	0.97	1.00		1.02	(0.76, 1.36)
**Sex**									
Men	Flooded		152	123	0.81	0.84	(0.65-1.08)	1.00	
	Non-flooded	0-2km	30,671	29,341	0.96	0.99	(0.96-1.02)	1.15	(0.90, 1.46)
		2-4km	24,246	23,490	0.97	1.00	(0.96-1.03)	1.22	(0.96, 1.56)
		4-6km	21,296	20,817	0.98	1.01	(0.97-1.04)	1.23	(0.96, 1.56)
		6-8km	17,992	17,520	0.97	0.99	(0.96-1.03)	1.28	(1.00, 1.63)
		8-10km	15,929	15,473	0.97	1.00		1.19	(0.92, 1.53)
Women	Flooded		180	146	0.81	0.90	(0.74-1.04)	1.00	
	Non-flooded	0-2km	33,687	32,621	0.97	1.02	(0.99-1.05)	1.17	(0.94, 1.45)
		2-4km	26,355	25,788	0.98	1.03	(0.99-1.06)	1.20	(0.96, 1.50)
		4-6km	23,756	22,904	0.96	1.00	(0.97-1.03)	1.14	(0.91, 1.43)
		6-8km	19,533	19,104	0.98	1.02	(0.99-1.05)	1.14	(0.91, 1.44)
		8-10km	17,618	16,861	0.96	1.00		1.11	(0.88, 1.40)
**Cause of death**									
Infectious	Flooded		0	0	-	-	(,)	-	(,)
	Non-flooded	0-2km	388	478	1.23	1.14	(0.88, 1.47)	1.00	
		2-4km	331	359	1.08	1.00	(0.77, 1.30)	0.83	(0.67, 1.03)
		4-6km	330	304	0.92	0.86	(0.66, 1.12)	0.76	(0.61, 0.95)
		6-8km	259	255	0.98	0.96	(0.73, 1.25)	0.78	(0.62, 1.01)
		8-10km	240	250	1.04	1.00		1.01	(0.97, 1.04)

	Boundary Area		Before (A)	After(B)	Ratio (B/A)	Before-after change ratio (95%CI) relative to 8-10 km		Before-after change ratio (95%CI) relative to flooded areas	
**Cause of death (Continued)**									
CVD	Flooded		142	107	0.75	0.78	(0.60, 1.03)	1.00	
	Non-flooded	0-2km	24,975	24,840	0.99	1.01	(0.98, 1.05)	1.29	(0.99, 1.66)
		2-4km	19,295	19,454	1.01	1.01	(0.98, 1.05)	1.35	(1.04, 1.74)
		4-6km	17,392	17,076	0.98	0.98	(0.66, 1.12)	1.28	(0.98, 1.66)
		6-8km	14,666	14,413	0.98	0.99	(0.95, 1.02)	1.27	(0.98, 1.65)
		8-10km	12,798	12,751	1.00	1.00		1.28	(0.97, 1.67)
Respiratory	Flooded		58	42	0.72	1.06	(0.70, 1.62)	1.00	
	Non-flooded	0-2km	11,612	8,343	0.72	1.03	(0.97, 1.09)	1.00	(0.67, 1.50)
		2-4km	9,234	6,642	0.72	1.03	(0.97, 1.08)	1.07	(0.71, 1.60)
		4-6km	8,012	6,054	0.76	1.06	(1.01, 1.12)	1.11	(0.73, 1.67)
		6-8km	6,682	5,029	0.75	1.05	(0.99, 1.11)	1.08	(0.71, 1.64)
		8-10km	6,114	4,353	0.71	1.00		0.94	(0.62, 1.44)
Mental	Flooded		3	11	3.67	2.45	(0.58, 10.31)	1.00	
	Non-flooded	0-2km	1,234	1,575	1.28	1.13	(0.97, 1.32)	0.35	(0.10, 1.30)
		2-4km	969	1,212	1.25	1.11	(0.96, 1.29)	0.31	(0.08, 1.20)
		4-6km	856	1,110	1.30	1.12	(0.96, 1.30)	0.37	(0.10, 1.42)
		6-8km	760	947	1.25	1.11	(0.96, 1.29)	0.24	(0.05, 1.22)
		8-10km	672	796	1.18	1.00		0.41	(0.10, 1.71)
External	Flooded		4	9	2.25	2.12	(0.59, 7.60)	1.00	
	Non-flooded	0-2km	1,914	1,861	0.97	0.99	(0.87, 1.12)	0.42	(0.13, 1.32)
		2-4km	1,498	1,546	1.03	1.09	(0.97, 1.24)	0.47	(0.14, 1.59)
		4-6km	1,310	1,238	0.95	0.99	(0.87, 1.12)	0.60	(0.17, 2.08)
		6-8km	1,124	1,053	0.94	0.97	(0.86, 1.10)	0.59	(0.16, 2.15)
		8-10km	1,081	1,027	0.95	1.00		0.47	(0.13, 1.69)
All others	Flooded		125	100	0.80	0.81	(0.62, 1.07)	1.00	
	Non-flooded	0-2km	24,235	24,865	1.03	0.99	(0.96, 1.03)	1.23	(0.95, 1.61)
		2-4km	19,274	20,065	1.04	0.99	(0.96, 1.03)	1.28	(0.98, 1.68)
		4-6km	17,152	17,939	1.05	1.00	(0.97, 1.04)	1.24	(0.95, 1.63)
		6-8km	14,034	14,927	1.06	1.01	(0.97, 1.04)	1.32	(1.01, 1.73)
		8-10km	12,642	13,157	1.04	1.00		1.23	(0.93, 1.62)
**Urban/rural**									
Urban	Flooded		204	169	0.83	0.84	(0.68, 1.04)	1.00	
	Non-flooded	0-2km	53,383	51,423	0.96	1.01	(0.99, 1.04)	1.14	(0.93, 1.40)
		2-4km	42,938	41,677	0.97	1.01	(0.99, 1.04)	1.20	(0.97, 1.48)
		4-6km	37,382	36,275	0.97	1.00	(0.97, 1.02)	1.19	(0.96, 1.46)
		6-8km	31,095	30,354	0.98	1.01	(0.98, 1.03)	1.22	(0.99, 1.50)
		8-10km	27,250	26,226	0.96	1.00		1.19	(0.96, 1.47)
Rural	Flooded		128	100	0.78	0.94	(0.70, 1.27)	1.00	
	Non-flooded	0-2km	10,975	10,539	0.96	1.01	(0.96, 1.06)	1.20	(0.92, 1.56)
		2-4km	7,663	7,601	0.99	1.03	(0.98, 1.08)	1.19	(0.90, 1.55)
		4-6km	7,670	7,446	0.97	1.01	(0.96, 1.06)	1.21	(0.92, 1.50)
		6-8km	6,430	6,270	0.98	1.00	(0.94, 1.05)	1.17	(0.88, 1.58)
		8-10km	6,297	6,108	0.97	1.00		1.06	(0.79, 1.43)
**Deprivation Quintile**									
Q1	Flooded		71	62	0.87	0.96	(0.65, 1.41)	1.00	
(least deprived)	Non-flooded	0-2km	14,377	13,752	0.96	1.01	(0.96, 1.07)	1.07	(0.75, 1.52)
		2-4km	9,791	9,505	0.97	1.01	(0.96, 1.06)	1.18	(0.83, 1.67)
		4-6km	8,396	8,193	0.98	1.00	(0.95, 1.05)	1.10	(0.77, 1.58)
		6-8km	7,766	7,629	0.98	1.03	(0.98, 1.08)	1.13	(0.78, 1.62)
		8-10km	6,057	5,798	0.96	1.00		1.04	(0.71, 1.53)

	Boundary Area		Before (A)	After(B)	Ratio (B/A)	Before-after change ratio (95%CI) relative to 8-10 km		Before-after change ratio (95%CI) relative to flooded areas	
**Deprivation Quintile (Continued)**									
Q2	Flooded		80	58	0.73	0.86	(0.57, 1.29)	1.00	
	Non-flooded	0-2km	12,775	12,364	0.97	1.02	(0.98, 1.08)	1.30	(0.92, 1.84)
		2-4km	9,177	8,926	0.97	1.02	(0.97, 1.08)	1.40	(0.99, 1.99)
		4-6km	9,245	8,844	0.96	1.00	(0.95, 1.05)	1.29	(0.91, 1.83)
		6-8km	8,109	7,940	0.98	1.01	(0.97, 1.07)	1.22	(0.85, 1.75)
		8-10km	6,844	6,525	0.95	1.00		1.17	(0.78, 1.76)
Q3	Flooded		73	79	1.08	1.14	(0.80, 1.61)	1.00	
	Non-flooded	0-2km	12,515	12,123	0.97	1.02	(0.97, 1.07)	0.89	(0.64, 1.23)
		2-4km	9,803	9,552	0.97	1.03	(0.98, 1.09)	0.92	(0.66, 1.29)
		4-6km	9,138	8,874	0.97	1.02	(0.97, 1.07)	0.92	(0.65, 1.29)
		6-8km	7,454	7,435	1.00	1.04	(0.99, 1.09)	1.02	(0.72, 1.44)
		8-10km	7,220	6,857	0.95	1.00		0.88	(0.62, 1.25)
Q4	Flooded		54	32	0.59	0.60	(0.38, 0.94)	1.00	
	Non-flooded	0-2km	12,108	11,526	0.95	1.02	(0.97, 1.07)	1.60	(1.02, 2.49)
		2-4km	10,008	9,893	0.99	1.02	(0.97, 1.07)	1.82	(1.15, 2.89)
		4-6km	9,015	8,835	0.98	1.01	(0.96, 1.06)	1.53	(0.97, 2.43)
		6-8km	7,637	7,405	0.97	1.01	(0.96, 1.06)	1.67	(1.06, 2.61)
		8-10km	7,446	7,166	0.96	1.00		1.67	(1.06, 2.64)
Q5	Flooded		54	38	0.70	0.79	(0.51, 1.21)	1.00	
(most deprived)	Non-flooded	0-2km	12,583	12,197	0.97	1.01	(0.95, 1.07)	1.37	(0.90, 2.08)
		2-4km	11,822	11,402	0.96	0.99	(0.94, 1.05)	1.37	(0.90, 2.10)
		4-6km	9,258	8,975	0.97	0.98	(0.93, 1.04)	1.43	(0.94, 2.19)
		6-8km	6,559	6,215	0.95	0.95	(0.90, 1.01)	1.32	(0.86, 2.02)
		8-10km	5,980	5,988	1.00	1.00		1.27	(0.82, 1.95)
**Place of death**									
Home	Flooded		65	49	0.75	0.77	(0.52, 1.14)	1.00	
	Non-flooded	0-2km	12,333	11,582	0.94	0.99	(0.94, 1.04)	1.21	(0.83, 1.78)
		2-4km	9,634	9,130	0.95	0.98	(0.93, 1.03)	1.26	(0.87, 1.84)
		4-6km	8,638	8,108	0.94	0.97	(0.92, 1.02)	1.24	(0.85, 1.83)
		6-8km	7,368	6,879	0.93	0.96	(0.92, 1.01)	1.27	(0.86, 1.86)
		8-10km	6,437	6,141	0.95	1.00		1.30	(0.88, 1.93)
Hospital	Flooded		178	134	0.75	0.80	(0.63, 1.01)	1.00	
	Non-flooded	0-2km	36,938	36,046	0.98	1.02	(0.99, 1.05)	1.26	(1.00, 1.58)
		2-4km	29,568	29,178	0.99	1.02	(0.99, 1.05)	1.36	(1.08, 1.72)
		4-6km	25,556	24,966	0.98	1.00	(0.98, 1.03)	1.30	(1.03, 1.64)
		6-8km	21,127	20,740	0.98	1.00	(0.98, 1.03)	1.28	(1.02, 1.62)
		8-10km	19,240	18,706	0.97	1.00		1.25	(0.98, 1.59)
Hospice	Flooded		19	12	0.63	0.68	(0.30, 1.54)	1.00	
	Non-flooded	0-2km	2,675	2,545	0.95	0.97	(0.87, 1.08)	1.38	(0.67, 2.84)
		2-4km	2,274	2,188	0.96	0.95	(0.86, 1.06)	1.38	(0.66, 2.91)
		4-6km	2,152	2,171	1.01	1.03	(0.93, 1.15)	1.50	(0.71, 3.21)
		6-8km	1,707	1,760	1.03	1.06	(0.95, 1.17)	1.26	(0.59, 2.70)
		8-10km	1,557	1,545	0.99	1.00		1.48	(0.65, 3.34)
Nursing home	Flooded		42	47	1.12	1.14	(0.72, 1.81)	1.00	
	Non-flooded	0-2km	6,133	5,962	0.97	1.03	(0.95, 1.11)	0.87	(0.57, 1.33)
		2-4km	4,525	4,586	1.01	1.07	(0.99, 1.15)	0.86	(0.56, 1.32)
		4-6km	4,441	4,576	1.03	1.08	(1.01, 1.17)	0.88	(0.57, 1.36)
		6-8km	3,638	3,726	1.02	1.06	(0.99, 1.14)	1.05	(0.68, 1.71)
		8-10km	3,124	3,019	0.97	1.00		0.88	(0.55, 1.40)

	Boundary Area		Before (A)	After(B)	Ratio (B/A)	Before-after change ratio (95%CI) relative to 8-10 km		Before-after change ratio (95%CI) relative to flooded areas	
**Place of death (Continued)**									
Residential	Flooded		19	15	0.79	1.03	(0.50, 2.13)	1.00	
home	Non-flooded	0-2km	4,600	4,269	0.93	1.06	(0.97, 1.16)	1.15	(0.58, 2.29)
		2-4km	3,362	2,958	0.88	1.01	(0.92, 1.10)	1.14	(0.57, 2.29)
		4-6km	3,145	2,829	0.90	1.02	(0.93, 1.11)	1.06	(0.52, 2.17)
		6-8km	2,733	2,613	0.96	1.09	(1,00, 1.19)	1.23	(0.59, 2.59)
		8-10km	2,394	2,104	0.88	1.00		0.97	(0.47, 2.00)
Other	Flooded		9	12	1.33	1.27	(0.50, 3.27)	1.00	
	Non-flooded	0-2km	1,679	1,558	0.93	0.86	(0.74, 0.99)	0.73	(0.30, 1.78)
		2-4km	1,238	1,238	1.00	1.01	(0.88, 1.17)	0.84	(0.34, 2.11)
		4-6km	1,120	1,071	0.96	0.92	(0.80, 1.06)	0.81	(0.34, 1.95)
		6-8km	952	906	0.95	0.89	(0.77, 1.03)	0.77	(0.31, 1.90)
		8-10km	795	819	1.03	1.00		0.79	(0.31, 2.02)

Appendix Table 4. Modification of covariates over time (10 years) in study period

Variables	RR	(95%CI)	p value
**Age**			
0-64	1.00		
65-74	0.76	(0.75, 0.76)	0.00
75+	1.09	(1.09, 1.10)	0.00
**Sex**			
Men	1.0		
Women	0.98	(0.98, 0.99)	0.00
**Deprivation quintile**			
Q1 (least deprived)	1.00		
Q2	0.95	(0.95, 0.96)	0.00
Q3	0.94	(0.93, 0.94)	0.00
Q4	0.92	(0.91, 0.93)	0.00
Q5 (most deprived)	0.90	(0.89, 0.91)	0.00
**Population density**			
Low (Q1)	1.00		
High (Q2-Q5)	0.99	(0.98, 1.00)	<0.01
**Region**			
North East	1.00		
North West	0.97	(0.96, 0.99)	0.00
Yorkshire and Hampshire	1.03	(1.01, 1.05)	0.00
East Midland	1.03	(1.02, 1.05)	0.00
West Midland	1.03	(1.01, 1.04)	0.00
East	1.04	(1.02, 1.06)	0.00
London	0.87	(0.85, 0.88)	0.00
South East	0.95	(0.94, 0.97)	0.00
South West	0.99	(0.98, 1.01)	0.39
Wales	1.00	(0.98, 1.01)	0.84
